# The Effect of *Lupinus albus* on Growth Performance, Body Composition and Satiety Hormones of Male Pigs Immunized against Gonadotrophin Releasing Factor

**DOI:** 10.3390/ani7030015

**Published:** 2017-03-02

**Authors:** Karen Moore, Bruce Mullan, Jae Cheol Kim, Frank Dunshea

**Affiliations:** 1 Faculty of Veterinary and Agricultural Sciences, The University of Melbourne, Parkville, Victoria 3010, Australia; fdunshea@unimelb.edu.au; 2 Grains and Livestock Industries, Department of Agriculture and Food Western Australia, South Perth, Western Australia 6151, Australia; bruce.mullan@agric.wa.gov.au (B.M.); jae.kim@abvista.com (J.C.K.)

**Keywords:** albus lupins, body composition, growth performance, immunocastrated male pigs, satiety hormones

## Abstract

**Simple Summary:**

Pigs immunized against gonadotrophin releasing factor (immunocastrates; IC males) have an increased feed intake, growth rate, back fat and fat deposition compared to entire males. A previous experiment found that *Lupinus albus* L. (albus lupins) has the potential to reduce feed intake and fat deposition in IC males. The current experiment aimed to develop a dietary management strategy using albus lupins for either 14 or 28 days pre-slaughter to reduce the increase in feed intake and subsequent increase in carcass fatness in IC males.

**Abstract:**

Two hundred and ninety four pigs were used with the aim to develop a dietary management strategy using *Lupinus albus* L. (albus lupins) to reduce the increase in feed intake and subsequent increase in carcass fatness in pigs immunized against gonadotrophin releasing factor (immunocastrates; IC males) and entire male pigs in the late finishing stage. From day (d) 0 to 28, IC males fed the control diet grew faster (*p* = 0.009) than entire males fed the control diet but there was no difference in growth rate between sexes for pigs fed albus lupins for 14 days pre-slaughter (Albus 14) or pigs fed albus lupins for 28 days pre-slaughter (Albus 28). From d 15 to 28, IC males receiving the Albus 14 diet grew more slowly (*p* < 0.001) than entire males receiving the Albus 14 diet. From d 15 to 28 (*p* < 0.001), IC males fed the control diet ate more feed than entire males fed the control diet, although there was no difference between sexes in feed intake of the Albus 14 and Albus 28 diet. Immunocastrates had a lower backfat when fed either Albus 14 or Albus 28 compared to the control diet, although there was no difference between diets for entire males. There was also a trend for pigs on the Albus 14 and Albus 28 diets to have a higher lean deposition (*p* = 0.055) and a lower fat deposition (*p* = 0.056) compared to the pigs on the control diet. Pigs fed the Albus 28 diet had a lower plasma ghrelin concentration compared to pigs fed the Albus 14 or the control diet (*p* = 0.002). Pigs fed the Albus 28 diet had a higher peptide YY concentration than those fed the control or albus 14 diet (*p* = 0.004). The inclusion of albus lupins at 20% in the diets of IC male pigs for either 14 or 28 days pre-slaughter was successful in reducing feed intake, body fat and backfat to similar levels of entire males. However, the growth rate of the IC male pigs was impacted more than would be desirable.

## 1. Introduction

*Lupinus albus* L. (albus lupins) have been reported to reduce feed intake in several pig experiments [1,2,3]. The most likely mechanism by which albus lupins reduce feed intake is by delayed transit in the stomach and small intestine. This delayed transit then feedbacks on satiety signals [1].

Moore et al. [3] investigated albus lupins inclusion in the diet of pigs immunized against gonadotrophin releasing factor (GnRF) in an attempt to reduce feed intake and fat deposition associated with the production of these pigs. However, the initial inclusion of albus lupins in Moore et al. [3] resulted in a greater reduction in feed intake than what was anticipated and so the concentration was reduced after the first week. For the final two-week period before slaughter, the feed intake and growth performance of immunocastrated (IC) male pigs on the albus lupin diet was similar to the entire males who received the control diet. The inclusion of albus lupins at 20% in the diet also appeared to have had some success in decreasing fat deposition in both entire males and IC males. For example, at a similar carcass weight, the back fat of IC males receiving the albus lupin diet was less than the back fat of entire males receiving the control diet (8.7 vs. 9.3 cm) [3]. Given the positive results, it is proposed to determine the effect of using albus lupins at a constant concentration on feed intake and fat deposition over a four-week period after the second immunization against GnRF.

The increase in fat deposition and feed intake of IC males occurs to a greater extent in the second two-week period after the second immunization against GnRF [3,4]. Therefore, it is proposed to investigate albus lupins inclusion in the final two-week period to minimize the increase in feed intake and fat deposition.

The aim of this project was to develop a dietary management strategy using albus lupins to reduce the increase in feed intake and subsequent increase in carcass fatness in IC male pigs. The hypotheses were (1) pigs immunized against GnRF which are fed a diet containing albus lupins for either 14 or 28 days prior to slaughter will have a reduced feed intake and growth rate compared to pigs immunized against GnRF receiving a standard finisher diet; (2) pigs fed albus lupins will have less fat compared to pigs receiving a standard finisher diet; (3) pigs immunized against GnRF and fed a diet containing albus lupins for either 14 or 28 days prior to slaughter will have a similar backfat compared to entire males receiving a standard finisher diet and; (4) pigs immunized against GnRF and fed albus lupins for 28 days prior to slaughter will have a lower overall daily feed intake but a similar fat composition compared to pigs immunized against GnRF and fed albus lupins for 14 days prior to slaughter.

## 2. Materials and Methods

The experimental protocol was approved by the Department of Agriculture and Food Western Australia’s Animal Research Committee and by the Animal Ethics Committee (Activity number 15-5-17). The animals were handled according to the Australian Code of Practice for the Care and Use of Animals for Scientific Purposes [5]. A total of 294 Large White × Landrace × Duroc entire male and immunocastrated male pigs were used in this experiment. The experiment was a 2 × 3 factorial with the main treatments being: (i) sex and lysine concentration [sex; entire males fed a diet with 0.64 g standardized ileal digestible (SID) lysine/MJ DE (mega joule digestible energy) for 28 days prior to slaughter (entire males) or IC males fed a diet with 0.64 g SID lysine/MJ DE for 14 days after second immunization against GnRF followed by 0.50 g SID lysine/MJ DE for 14 days prior to slaughter (IC male)]; and (ii) feeding strategy [control; 200 g/kg albus lupins for 28 days prior to slaughter (Albus 28); or control diet for 14 days after second immunization against GnRF followed by 200 g/kg albus lupins for the last 14 days prior to slaughter (Albus 14)].

### 2.1. Allocation and Housing

Two hundred and ninety four entire male pigs were sourced from a high health status commercial herd at 39.4 ± 2.79 kg liveweight (LW). Upon arrival, the pigs were individually identified with ear tags, weighed and stratified on their LW. The pigs allocated to being immunocastrated received a priming dose of an anti-gonadotrophin releasing factor immunological product (Improvac^®^, Zoetis Australia, Rhodes, Australia) on day (d) −28 (where d 0 is when all pigs received the second dose of the anti-gonadotrophin releasing factor vaccine). The entire males did not receive a placebo injection. The pigs were group housed (*n* = 7) in a naturally ventilated grower/finisher shed and they had ad libitum access to water and a commercial feed via a single-spaced feeder.

### 2.2. Diets and Feeding Regime

On d 0, all pigs received the experimental diet and pigs who had received the priming dose of the anti-gonadotrophin releasing factor vaccine were given their second dose. The experimental diets were formulated to the same nutrient specifications (13.5 MJ DE and 0.64 g standardized ileal digestible lysine (SID)/MJ DE (high) or 0.50 g SID/MJ DE (low)). The diets were formulated so that the IC male pigs were fed as entire males for 2 weeks (from d 0; high) and then the lysine concentration in the diet was reduced for the remaining 2 weeks (low; based on a previous lysine requirement study for IC males of this particular genetics; Moore et al. [6]). The entire male pigs continued to receive the diet adequate for an entire male pig (high). The composition of the experimental diets is given in Table 1. The diets were also analyzed for quantitative AA composition (Australian Proteome Analysis Facility, Sydney, NSW, Australia) and the results are presented in Table 2. The diets fed for each sex and feeding strategy for d 0 to 14 and d 15 to 28 are described in Table 3.

### 2.3. Growth Performance

Pigs were weighed weekly and feed intake was determined on day 0, 7, 14, 21 and 28 to measure average daily gain and voluntary feed intake. The feed conversion ratio was calculated on a weekly basis from when the feeding of the experimental diets commenced.

### 2.4. Dual X-Ray Absorptiometry

Twelve pigs per treatment (three pigs/pen randomly selected from four replicate pens, so 72 in total (12 pigs × 6 treatments)) were scanned on day 27 using dual-energy x-ray absorptiometry (DXA) to determine the percentage of bone mass composition, lean and fat. The pigs were removed from feed and fasted for approximately 16 h before scanning. Immediately before scanning, the pigs were weighed and then transferred to the DXA facility. They were injected intramuscularly with Stresnil^®^ (azaperone 40 mg/mL, Stresnil Neuroleptic Injection for Pigs, Ausrichter Pty Ltd., Newtown, NSW, Australia) at 2 mL/10 kg LW. When sufficiently sedated, the pigs were transferred to the DXA machine (Norland XR46 Densitometer Machine, Norland Products Inc., Cranbury, NJ, USA) [7]. The pigs were scanned in ventral-recumbency, with hind legs extended and forelegs positioned caudally. Whole body mode was used to scan and the scan was subsequently analyzed using whole body analysis. Measurements made by DXA included lean tissue mass, fat tissue mass and bone mineral content. After scanning, the pigs were placed in a recovery room until they were able to stand and were then returned to their pens. The pigs were given their respective diets on return to their pens.

### 2.5. Slaughter Procedure

Four weeks after the diets were introduced, the pigs were individually tattooed, removed from feed overnight and transported to a commercial abattoir (approximately 90 min transport time). The pigs were stunned using a carbon dioxide, dip-lift stunner set at 85% CO_2_ for 1.8 min (Butina, Denmark). Exsanguination, scalding, dehairing and evisceration were performed using standard commercial procedures. Hot carcass weight (HCW, AUSMEAT Trim 13; head off, fore trotters off, hind trotters on; AUS-MEAT Ltd, South Brisbane, Qld, Australia) and P2 backfat depth, 65 mm from the dorsal midline at the point of the last rib (PorkScan Pty Ltd., Canberra, Australia) were measured approximately 35 min after exsanguination, prior to chiller entry (2 °C, airspeed 4 m/s).

### 2.6. Satiety Hormones

Blood samples (20 mL in lithium heparin tubes) were collected on d 14 and 28 from the same pigs that were selected for DXA scanning. The blood samples were centrifuged at 2000× *g* for 15 min to recover plasma and were stored at −20 °C until analyzed. Plasma insulin, peptide tyrosine tyrosine (peptide YY), cholecystokinin (CKK), glucagon-like peptide 1 (GLP-1) and ghrelin were quantified using commercial kits (Mercodia Porcine Insulin ELISA 10-1200-01, Sapphire BioSciences Pty Ltd. (Redfern, Australia); Pig Peptide tyrosine tyrosine, PYY ELISA Kit MyBioSource MBS903317, Resolving Images Pty Ltd. (Melbourne, Australia); Porcine Cholecystokinin (CKK) ELISA kit MyBioSource MBS264395, Resolving Images Pty Ltd.; Pig glucagon-like peptide 1, GLP1 ELISA kit, MyBioSource MBS943508, Resolving Images Pty Ltd. and; Porcine Ghrelin (GHRL) ELISA kit MyBioSource MBS2019385, Resolving Images Pty Ltd.; respectively).

### 2.7. Statistical Analysis

General analysis of variance was performed with the GENSTAT 18 program (VSN International Ltd., Hemel Hempstead, UK) to analyze the main effects of sex and lysine concentration and diet on growth performance, carcass quality, body composition and satiety hormones. For growth performance and carcass data, the pen was the experimental unit. For the DXA and satiety hormone measures, pig was the experimental unit. Repeated measures analysis of variance was used to analyze the satiety hormones. A level of probability of < 0.05 was used to determine statistical difference between the means. A level of probability of < 0.1 but > 0.05 was determined to be a trend. Fisher’s-protected least significant differences were used to determine differences among treatments.

## 3. Results

### 3.1. Growth and Carcass Performance

Immunocastrated males grew faster (*p* = 0.005), ate more feed (*p* = 0.046) and had a better feed conversion (*p* = 0.032) compared to entire males between d 0 and 14. Pigs fed Albus 28 had a lower daily gain (*p* < 0.001), lower feed intake (*p* < 0.001) and tended to have a poorer feed conversion ratio (*p* = 0.077) compared to pigs fed Control or Albus 14 from d 0 to 14. From d 0 to 28, there was trend for pigs fed Albus 14 to have a poorer feed conversion (*p* = 0.091) than those fed either the Control diet or Albus 28 (Table 4).

From d 15 to 28, IC males receiving either Albus 14 or Albus 28 grew slower (*p* < 0.001) than entire males receiving Albus 14 or Albus 28, however IC males on the control diet grew faster than entire males on the Control diet. From d 0 to 28 (*p* = 0.009), there was a sex by feeding strategy interaction in that IC males fed the Control diet grew faster than entire males fed the Control diet but there was no difference in growth rate between sexes for either Albus 14 or Albus 28. There was a sex by feeding strategy interaction for feed intake for d 15 to 28 (*p* < 0.001) and d 0 to 28 (*p* = 0.009) where IC males fed the Control diet ate more feed than entire males fed the Control diet, however, there was no difference between sexes in feed intake of the Albus 14 and Albus 28 diet. There was a sex by feeding strategy interaction for the feed conversion ratio from d 15 to 28 (*p* = 0.034) where IC males fed Albus 14 had a worse feed conversion ratio compared to those fed either Albus 28 or the Control diet. There was no difference between feeding strategies for the entire males. 

There was a sex by feeding strategy interaction (*p* = 0.027) for carcass weight where IC males fed either Albus 14 or Albus 28 had a lower carcass weight compared to those on the Control diet, however, there was no difference between feeding strategies for entire males. There was also a sex by feeding strategy interaction (*p* = 0.028) for dressing percentage where IC males fed Albus 28 had a lower dressing percentage compared to the other feeding strategies, however, there was no difference between diets for entire males. There was a sex by feeding strategy interaction (*p* = 0.042) for backfat where IC males had a lower backfat when fed either Albus 14 or Albus 28 compared to the Control diet, however, there was no difference between feeding strategies for entire males.

### 3.2. Body Composition

There was a sex by feeding strategy interaction (*p* = 0.001) for percentage bone mineral content (BMC) in that IC males on the Control diet had a lower BMC than those fed Albus 28, however entire males on the Control diet had a higher BMC than those fed Albus 28. Immunocastrated males had a lower percentage lean mass (*p* < 0.001) and a higher percentage fat deposition (*p* < 0.001) compared to entire males. There was a trend for pigs fed Albus 14 and Albus 28 to have a higher lean deposition (*p* = 0.055) and a lower fat deposition (*p* = 0.056) compared to the pigs on the Control diet. There were no interactions for lean deposition or fat deposition (Table 5).

### 3.3. Satiety Hormones

**Figure 1:**
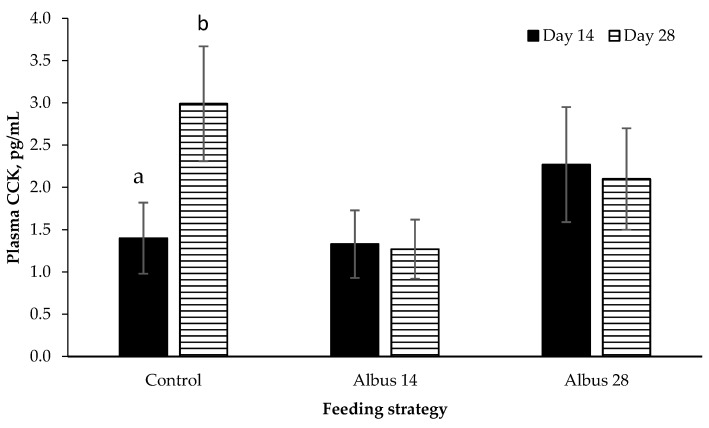
Plasma cholecystokinin (CYY) concentration on day 14 and day 28 after the implementation of the feeding strategy (mean ± SE; *n* = 24). There was a time × diet interaction (*p* = 0.002). Data for entire males and IC males have been pooled as there was no effect of sex or any interactions (*p* > 0.05). ^a,b^ Different superscripts within diets are significantly different.

There was no main effect of sex or feeding strategy on plasma CKK concentration (*p* > 0.05; Figure 1). There was a time × feeding strategy interaction where plasma CKK concentration increased from d 14 to d 28 for pigs fed the Control, however there was no change from d 14 to d 18 for pigs on Albus 14 or Albus 28 (*p* = 0.002).

The majority of pigs (95%) had GLP-1 concentrations below the level of detection. There was no effect of sex, feeding strategy, time or any interactions for plasma glucagon-like peptide 1 (*p* > 0.05, data not shown).

**Figure 2:**
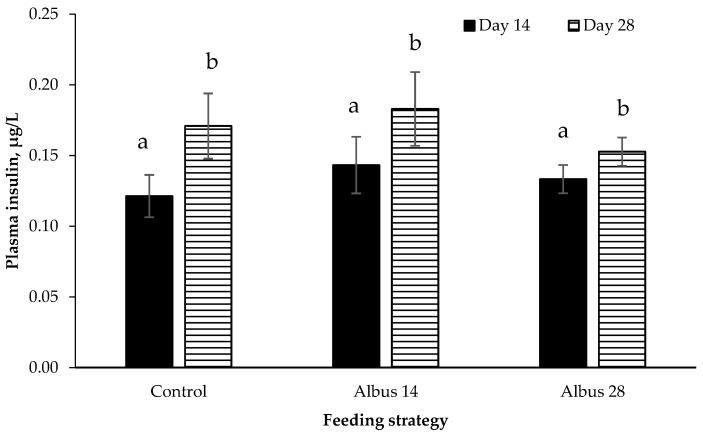
Plasma insulin concentration on day 14 and day 28 after the after the implementation of the experimental diets (mean ± SE; *n* = 24). Plasma insulin increased with time (*p* = 0.037). Data for entire males and IC males have been pooled as there was no effect of sex or any interactions (*p* > 0.05). ^a,b^ Different superscripts within diets are significantly different.

There was no effect of sex or feeding strategy or any interactions on plasma insulin concentration (*p* > 0.05). Plasma insulin concentration increased from d 14 to d 28 (*p* = 0.037; Figure 2).

**Figure 3:**
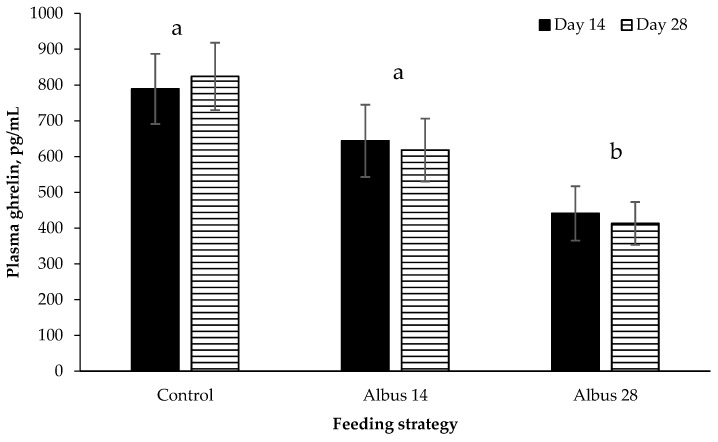
Plasma ghrelin concentration on day 14 and day 28 after the implementation of the experimental diets (mean ± SE; *n* = 24). There was a significant effect of diet (*p* = 0.002). Data for entire males and IC males have been pooled as there was no effect of sex or any interactions (*p* > 0.05). ^a,b^ Different superscripts between diets are significantly different.

There was no effect of sex or time or any interactions on plasma ghrelin concentration (*p* > 0.05; Figure 3). Pigs fed Albus 28 had a lower plasma ghrelin concentration compared to pigs fed the Control or Albus 14 (*p* = 0.002). 

**Figure 4:**
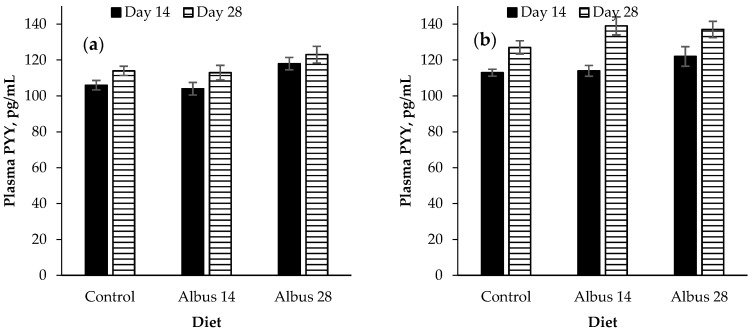
Plasma peptide YY (PYY) concentration on day 14 and day 28 after the implementation of the experimental diets for (**a**) entire male pigs and (**b**) immunocastrated male pigs (mean ± SE; *n* = 12). The *p*-values for sex, diet, time and time by sex were *p* < 0.001, *p* = 0.004, *p* < 0.001 and *p* = 0.002, respectively. There were no other significant interactions (*p* > 0.05).

Immunocastrated male pigs had a higher plasma PYY concentrations than entire male pigs (*p* < 0.001, Figure 4). Pigs fed Albus 28 had a higher PYY concentration than those fed the Control diet or Albus 14 (*p* = 0.004). Peptide YY concentration increased from d 14 to d 28 (*p* < 0.001). There was a time × sex interaction where there was no difference in PYY concentration for entire males from d 14 to d 28, however, the PYY of IC males increased from d 14 to d 28 (*p* = 0.002). There were no other interactions (*p* > 0.05).

## 4. Discussion

The hypothesis that pigs immunized against GnRF which are fed a diet containing albus lupins for either 14 or 28 days prior to slaughter will have a reduced feed intake and growth rate compared to pigs immunized against GnRF fed a standard finisher diet was supported. The feed intake of IC males was 23% less for pigs on the Albus 28 diet and 20% less for the Albus 14 diet compared to the IC males receiving the control diet. The growth rate was 23% less for IC males on the Albus 28 diet and 24% less for those fed the Albus 14 diet compared to control. The reduction in feed intake and growth rate of pigs fed albus lupins concurs with Moore et al. [3]. Albus lupins are thought to decrease feed intake by delayed transit through the stomach and small intestine. This may then feedback through satiety signals [1].

Although there was a reduction in feed intake as expected when the IC male pigs were fed albus lupins, the reduction was greater than anticipated (approximately 25% compared to the predicted 15%). The analyzed standardised ileal digestible lysine levels of the diets were as estimated and the IC males that received the control diet with the low lysine concentration consumed the feed and grew as expected. It appears that the largest decrease in feed intake was associated with the albus low lysine diet (diet received from d 15 to 28 for the IC males). It is suggested that perhaps there was increased acceptability issues with the albus low diet (associated with the albus lupins rather than the lysine concentration) in older or heavier pigs which was not expected and it is unknown why this may have been the case. Perhaps this is related to the large increase in feed intake that is generally observed in IC males around 2 weeks after secondary immunization that could not be exhibited in pigs that had only just been introduced to the albus diet.

The increased reduction in feed intake affected the daily gain of IC males to a greater extent than entire males. This was probably because the entire males have faster and leaner growth than IC males at a similar level of energy intake as they have a greater capacity for lean tissue growth [8]. 

The desired outcome was for the feed intake and backfat of IC males to be similar to that of entire males whilst maintaining a slight improvement in growth rate of the IC males. However, from d 15 to 28, the daily gain, feed intake and feed conversion of the IC males on the albus diets were lower than the entire males on the control diet. In contrast, Moore et al. [3] found that the IC males fed a 20% albus lupin diet had a similar daily gain, feed intake and feed conversion ratio compared to entire males fed the control diet from d 15 to 28. The differences between the two experiments may be due to acceptability issues of the albus low diet in the current experiment. Due to the inconsistent results, it is suggested that further work on including albus lupins in the diet be conducted. This should include titrating the inclusion level of albus lupins to ensure they are included at the appropriate rate to maximize growth performance whilst reducing feed intake of the IC males.

The performance of pigs in this research facility is often superior to that observed in commercial production systems due to its very high health status and so the impact of the albus lupin diets on growth rate and feed intake are discussed further in relation to previous experiments conducted in this facility using IC male pigs of the same genotype and similar liveweights. The average daily gain and feed intake of IC male pigs fed the equivalent of a control diet ad libitum in this research facility for the second two-week period after the second immunization against GnRF are 1.18 kg/d and 3.54 kg/d, respectively [3,4,6]. In the current experiment, IC male pigs fed either the Albus 14 or Albus 28 diet from d 15 to 28 had a 38% and 15% lower daily gain and 30% and 23% lower feed intake, respectively, compared to the standard growth rates and feed intakes of IC males fed a control diet in this research facility. No measures of welfare, pig behavior and hunger were incorporated in the current experiment, so definitive conclusions on the possible impact of the reduction in feed intake beyond the reduction in growth rate on welfare cannot be drawn.

The reduction in the feed intake of IC male pigs fed Albus 14 from d 15 to 28 was equivalent to a restriction of approximately 2.5 times maintenance (calculated using the equation MEm (kJ/d) = 444 kJ × BW^0.75^, where MEm = metabolic energy maintenance and BW = liveweight [9]). Moore et al. [4] restricted individually housed IC males to 2.5 times maintenance by restricting the amount of feed fed to the pigs. At similar liveweights and hence feed intakes, the growth rate was similar to that of the IC males fed Albus 14 from d 15 to 28 in the current experiment (0.80 vs. 0.73 kg/d, respectively). 

This experiment used a similar strategy as qualitative restriction to restrict feed intake in an attempt to reduce backfat and increase carcass leanness. Qualitative restriction refers to offering feed ad libitum but reducing feed quality (for example by including bulky ingredients containing dietary fibre or with non-fibrous nutrients known to suppress appetite) [10,11]. Less energy is consumed from low-quality food by ad libitum fed pigs so intake is restricted [12,13]. An alternative strategy is to restrict feed intake by restricting the amount of feed (quantitative restriction). Restrictively fed IC male pigs have been found to have a reduced backfat [14] and increased carcass leanness [15]. However, in group-housed pigs, restricting the feed intake by restricting the amount of feed has welfare issues in terms of increased aggression [15]. Other researchers have also noted that quantitative restriction is also associated with signs of hunger (review by Tolkamp and D’Eath [16]).

D’Eath et al. [17] and Tolkamp and D’Eath [16] reviewed the two methods of restricted feeding and conclude that there is controversy on the welfare benefits of qualitative versus quantitative restriction. Some researchers have concluded that qualitative feed restriction has welfare advantages over quantitative feed restriction because it promotes satiety and more normal feeding behavior [16]. In contrast, other researchers conclude that there are no welfare improvements in quantitative versus qualitative restriction because the pigs are still experiencing ‘metabolic hunger’. Tolkamp and D’Eath [16] and D’Eath et al. [17] conclude that the differences between researchers can be attributed to (i) the methodologies used to measure animal hunger and their perceived value; (ii) assumptions about what controls food intake and feeding behavior and (iii) how ‘naturalness’ of behavior is weighted as a determinant of animal welfare. In the present experiment, measures of animal welfare were not incorporated and it is suggested that further research using albus lupins to reduce feed intake incorporate some measures of welfare and animal hunger. However, as noted by Tolkamp and D’Eath [16], better methodologies to measure animal hunger and a greater understanding of what is controlling feed intake may be required for this to be effective.

The hypothesis that pigs fed albus lupins will have less fat compared to pigs receiving a standard finisher diet was supported. Pigs on both of the albus lupin diets had approximately 2.5% less body fat and 0.9 mm lower backfat compared to pigs fed the control diet. This concurs with findings from Quiniou et al. [14] who when restricting feed intake to 2.5 or 2.75 kg/d, which equated to 15% and 22% lower feed intake than ad libitum, found that backfat thickness was reduced by between 0.6 and 1.0 mm in the restricted fed pigs compared to the ad libitum fed pigs. Van Nevel et al. [2] and Moore et al. [3] also found that backfat was reduced when albus lupins were included in diets. The reduced backfat and an increase in lean in van Nevel et al. [2] was attributed to the slower growth rate of the pigs fed albus lupins. 

The reduction in body fat percentage and backfat for pigs fed the albus lupin diets was greater for IC males compared to entire males. This is likely because of the increased fat deposition associated with IC males two weeks after the second immunization against GnRF, as demonstrated by Moore et al. [3] and Moore et al. [4].

The hypothesis that pigs immunized against GnRF and fed a diet containing albus lupins for either 14 or 28 days prior to slaughter will have a similar backfat compared to entire males receiving a standard finisher diet was supported in principle as the *p*-value was 0.042 which can be considered equivocal. When combined with the lower body fat percentage and decreased feed intake, this provides further support in principle for albus lupins to be included in the diet of IC males in markets where producers are penalized for excessive back fats provided the large decrease in growth rate can be overcome. Moore et al. [3] found that IC male pigs fed albus lupins for 28 days had a similar backfat to entire males fed the control diet for 28 days. Further work may be required to confirm if the reduction in backfat of IC males fed diets containing albus lupins for either 14 or 28 days prior to slaughter to similar levels as entire males fed a standard finisher diet is a real effect.

The hypothesis that pigs immunized against GnRF and fed albus lupins for 28 days prior to slaughter will have a lower overall daily feed intake but a similar fat composition compared to pigs immunized against GnRF and fed albus lupins for 14 days prior to slaughter was also supported. Immunocastrated male pigs fed albus lupins for 28 days ate 5% less feed overall than IC males fed albus lupins for 14 days pre-slaughter, while their percentage fat composition was similar. However, when the feed intake was compared over the period between d 15 and 28 only, IC males that were fed albus lupins for this period only (Albus 14) had a 9% lower feed intake than those that had received albus lupins for the entire 28 days (Albus 28). Therefore, the albus lupin diet would only need to be fed for the final two-week period before slaughter to minimize fat deposition and the increase in backfat provided, as mentioned already, decrease in growth rate can be alleviated. This could possibly be achieved with a lower inclusion rate of albus lupins.

Pigs on the albus feeding strategies tended to have a higher percentage lean mass compared to those on the Control. This is supported by van Nevel et al. [2] who found that when albus lupins were included at 30%, there was a tendency for the percentage of lean content to increase. The trend for an increase in lean content and a decrease in fat content is most likely associated with the decrease in feed intake of the pigs fed albus lupins resulting in a decreased growth rate. In contrast, when Quiniou et al. [14] restrictively fed pigs by reducing the amount of feed, there was no difference in lean meat when using an X-ray computed tomography scanner to measure the volumetric lean content in half carcasses. The growth rate was also decreased when feed intake was reduced by approximately 22% [14].

There was no effect of albus lupins on dressing percentage in this experiment. In contrast, other researchers have found a decrease in dressing percentage when pigs were fed diets containing varying concentrations of albus lupins [1,2,18]. The decrease in dressing percentage supports the theory of the delayed transit of the albus lupins through the stomach and small intestine [1]. 

Several satiety hormones were also investigated to try to determine how the albus lupins were reducing feed intake. Gut hormones such as CCK and GLP-1 and stomach distension are short-term signals which have a direct effect on gastric emptying and meal termination. Signals that work in the long term determine the sensitivity to these short-term signals. This includes hormones such as leptin, insulin, PYY and ghrelin. These hormones have longer-lasting postprandial effects on meal initiation and satiety [19]. The consistent results in satiety hormones across a wide range of mammals suggest that outcomes from human studies are relevant to feed consumption in agricultural animals and gastrointestinal hormone functions [20] and therefore some of the results are discussed in relation to this. 

There was a time by feeding strategy interaction where plasma CCK concentration increased from d 14 to d 28 for pigs fed the control diet, however there was no change from d 14 to d 28 for pigs on Albus 14 or Albus 28. Pigs fed the diets containing albus lupins had a lower feed intake than those on the control diet for the period d 15 to 28 after the second immunization against GnRF. Given that CCK is a short-term signal that determines meal termination [19], perhaps the higher concentrations of CCK observed in the control diet occur to curb food consumption. In comparison, the feed intake of those pigs fed the albus diets had already decreased and therefore a longer acting hormone is contributing to the decrease in food intake. Cholecystokinin is released into the blood when carbohydrates, fats and protein are present in the duodenum [21,22,23]. It is one determinant of meal termination, with a necessary condition for the appetite-suppressing effect of CCK being a full stomach [19]. Cholecystokinin is thought to have a peripheral effect on the induction of satiety, with one means being the inhibition of gastric emptying [24]. However, other work has suggested that this is unlikely to be the main mechanism by which CCK affects satiety [25]. 

In the current experiment, there was no diet or sex effect on GLP-1 or insulin. Glucagon-like peptide-1 is produced by endocrine L-cells which are mostly found in the distal ileum and colon [26,27]. Therefore, it is suggested that the effect the albus lupins are having on decreasing food intake was independent to the GLP-1 signaling system. Glucagon-like peptide-1 is also thought to stimulate insulin secretion [1,28] and as there was no effect of GLP-1, it is not unexpected that there was also no difference in insulin levels between diets. 

Plasma ghrelin was decreased in pigs fed Albus 28 compared to the Control and Albus 14. Ghrelin is secreted in the stomach and stimulates eating with concentrations decreasing following meals [20]. Although ghrelin is secreted in the stomach, the stomach does not appear to contain the sensing mechanisms that suppress ghrelin secretion after meals [20]. The signals which suppress ghrelin appear to originate further in the small intestine [29]. The decrease in ghrelin in pigs receiving Albus 28 concurs with the suggestion by Dunshea et al. [1] that albus lupins possibly feedback on satiety signals by delayed transit in the stomach and small intestine. However, it does not explain why there was no difference in ghrelin concentration between d 14 and d 28 in pigs fed Albus 14 as up until d 14 these pigs had received the control diet. 

There was a time × sex interaction for peptide YY where there was no difference in PYY concentration for entire males from d 14 to d 28, however, the PYY of IC males increased from d 14 to d 28. This is opposite to what would be expected as an increase in peptide-YY would traditionally be associated with a decrease in food intake, but in this experiment the IC males had a higher feed intake compared to the entire males. It is proposed that perhaps the increase in concentration was not enough of an increase as it has been found that significant reductions in food intake were only associated with PYY concentrations which were higher than a high-calorie meal [20]. 

Pigs fed Albus 28 had a higher plasma PYY concentration than those fed the control or Albus 14. The feed intake of pigs fed both Albus 28 and Albus 14 was reduced on d 28 compared to the control diet. It would be expected that the PYY concentration of pigs fed Albus 14 would also be higher on d 28 but this was not observed and may suggest that other satiety hormones are playing a larger role in decreasing the feed intake of pigs fed albus lupins.

Peptide YY is produced and secreted in the distal ileum and colon from the endocrine L-cells which also express GLP-1 [20]. Although peptide-YY and GLP-1 are secreted from the same cells, their plasma concentrations can be a different pattern to their bioactive forms because dipeptidyl peptidade-IV, which is found circulating in plasma, activates PYY but inactivates GLP-1 [20]. This may partly explain why there was a difference in PYY concentrations but not GLP-1 concentrations in this experiment. Peptide YY is one of the hormones which play a role in the ileal brake mechanism [20]. The ileal brake refers to the feedback mechanism which ensures nutrient digestion and absorption is optimized by controlling the transit of a meal through the gastrointestinal tract [30].

Further study is required to clarify how albus lupins are depressing feed intake. The delayed passage of the albus lupins through the digestive tract, as observed by Dunshea et al. [1], is resulting in a long-term depression of feed intake due to the action of hormones such as peptide YY and ghrelin which are released by the digestive tract [31]. However, there may also be a combination of other factors in play such as excessive volatile fatty acid production in the hindgut and the presence of saponins which have bitter and astringent characteristics which may inhibit feed intake and/or increase retention time [1].

## 5. Conclusions

The inclusion of albus lupins at 20% in the diets of IC male pigs was successful at reducing feed intake, body fat and backfat to similar levels of entire males. However, the growth rate of the IC male pigs was impacted more than would be desirable. In comparison, in Moore et al. [3] where albus lupins were included at 20% for the last 14 days pre-slaughter, the feed intake, growth rate and backfat of IC males were similar to that of entire males. Due to the inconsistent results on the growth rate of IC males, further investigation on the effect of albus lupins in the diets of immunocastrated male pigs is warranted. It is suggested that the effect of albus lupins on growth performance and backfat be further investigated using titrated levels of albus lupins, for example, from 10% to 20% to determine an appropriate level to include in order to maximize the decrease in feed intake and fat deposition whilst minimizing the effect on growth rate. If possible, measures of welfare and hunger should also be included to determine the effect that the qualitative feed restriction is having.

## Figures and Tables

**Table 1:** Composition of the experimental diets.

Ingredients g/kg, as-Fed	Control Low ^1^	Control High ^2^	Albus Low ^1^	Albus High ^2^
Barley	400	400	400	579
Wheat	384	257	260	101
Mill run	50	85	91	10
Lupins, angustifolius	100	100	0	0
Lupins, albus	0	0	200	200
Canola meal	20	100	10	49
Soybean meal	10	10	10	14.5
Bloodmeal	1.28	15.2	2	20
Tallow	15.3	15.7	10	10
Limestone	11.1	11.1	11.5	11.0
DiCal Phosphorus	1.31	0	0	0
Salt	2	2	2.48	2
l-Lysine HCL	2.43	2.29	0.61	0.83
Methionine	0.51	0.56	0	0.70
Phytase ^3^	0.20	0.20	0.20	0.20
Choline chloride, 60%	1.02	0.32	1.76	1.53
Vitamins and minerals ^4^	1.0	1.0	1.0	1.0
Nutrient Composition ^5^				
DE, MJ/kg	13.5	13.5	13.5	13.5
CP, g/kg	144	176	172	190
Ca, g/kg	8.00	8.00	8.00	8.00
Total P, g/kg	6.35	6.85	6.53	6.25
Available P, g/kg	4.50	4.50	4.49	4.50
NDF, g/kg	175	196	188	182
ADF, g/kg	53.3	49.5	59.2	64.8
g SID Lys/MJ DE ^6^	0.50	0.64	0.50	0.64

^1^ Low—lysine concentration was 0.50 g standardized ileal digestible lysine/MJ DE, fed to immunocastrated males for 14 days prior to slaughter; ^2^ High—Lysine concentration was 0.64 g standardized ileal digestible lysine/MJ DE, fed to entire males for entire 28 days prior to slaughter and to immunocastrated males for 14 days after the second immunization against GnRF; ^3^ Phytase from Phyzyme, Danisco Australia Pty Ltd. (Banksmeadow, Australia); ^4^ Provided per kg of final diet: 7000 IU Vitamin A, 1400 IU Vitamin D3, 20 g Vitamin E, 1 g Vitamin K, 1 g Vitamin B1, 3 g Vitamin B2, 1.5 g Vitamin B6, 15 mg Vitamin B12 12 g niacin, 10 mg pantothentic acid, 0.19 g folic acid, 30 mg biotin, 10.6 g Calcium pantothenatic, 60 g iron, 100 g zinc, 40 g manganese, 10 g copper, 0.2 g cobalt, 0.5 g iodine, 0.3 g selenium, and 20 g antioxidant.; ^5^ Calculated composition.; ^6^ SID: standardized ileal digestible lysine/MJ digestible energy.

**Table 2:** Quantitative amino acid analysis of the diets.

Amino Acid g/kg, as-Fed	Control Low ^1^	Control High ^2^	Albus Low ^1^	Albus High ^2^
Histidine	3.6	4.7	3.7	4.9
Isoleucine	5.6	6.4	6.2	6.9
Leucine	10.1	12.7	11.1	14.2
Lysine	7.4	9.2	7.2	9.6
Methionine	1.8	2.4	1.6	1.9
Phenylalanine	6.7	8.2	7.0	9.1
Threonine	5.1	6.5	5.7	7.1
Valine	7.0	9.3	7.6	10.2
Alanine	5.5	7.7	5.9	8.1
Arginine	9.5	10.8	11.9	12.3
Aspartic acid	10.5	12.5	12.4	14.8
Glycine	6.3	8.8	6.8	8.4
Glutamic acid	34.0	37.4	35.1	38.1
Proline	11.1	13.6	10.7	14.0
Serine	6.8	8.0	7.6	8.7
Tyrosine	2.8	3.5	3.7	4.0

^1^ Low—lysine concentration was 0.50 g standardized ileal digestible lysine/MJ DE; ^2^ High—Lysine concentration was 0.64 g standardized ileal digestible lysine/MJ DE.

**Table 3:** Diets fed to each sex and feeding strategy for the periods day 0 to 14 and day 15 to 28.

Sex	Day 0 ^1^ to 14	Day 15 to 28	Number of Pens/Treatment ^2^
Feeding Strategy			
**Entire males**			
Control	Control high ^3^	Control high	7
Albus 14	Control high	Albus high	7
Albus 28	Albus high	Albus high	7
**Immunocastrated males**			
Control	Control high	Control low ^4^	7
Albus 14	Control high	Albus low	7
Albus 28	Albus high	Albus low	7

^1^ Day 0 = second immunization against GnRF given to immunocastrated males; ^2^ 7 pigs/pen; ^3^ High—lysine concentration was 0.64 g standardized ileal digestible lysine/MJ DE; ^4^ Low—lysine concentration was 0.50 g standardized ileal digestible lysine/MJ DE.

**Table 4:** Growth and carcass performance for entire male and immunocastrated male pigs fed three different feeding strategies from 72.3 to 101.1 kg liveweight (*n* = 7).

	Entire Male			Immunocastrated Male			SED ^d^	*p*-Value		
	Control ^a^	Albus 14 ^b^	Albus 28 ^c^	Control	Albus 14	Albus 28		Sex	Feeding Strategy	Sex × Feeding Strategy
**Daily gain (kg/day)**										
d 0–14	1.11	1.04	0.861	1.18	1.14	0.950	0.050	0.005	<0.001	0.93
d 15–28	1.12 ^j^	0.929 ^i^	1.09 ^j^	1.28 ^k^	0.726 ^h^	0.929 ^i^	0.060	0.062	<0.001	<0.001
d 0–28	1.11 ^i^	0.985 ^h^	0.974 ^h^	1.23 ^j^	0.933 ^h^	0.940 ^h^	0.040	0.67	<0.001	0.009
**Feed intake (kg/day)**										
d 0–14	2.72	2.64	2.29	2.78	2.83	2.34	0.084	0.046	<0.001	0.42
d 15–28	3.05 ^i^	2.49 ^h^	2.72 ^h^	3.86 ^j^	2.49 ^h^	2.74 ^h^	0.134	0.001	<0.001	<0.001
d 0–28	2.88 ^i^	2.57 ^h^	2.51 ^h^	3.32 ^j^	2.67 ^h^	2.54 ^h^	0.094	0.001	<0.001	0.009
**Feed conversion ratio**										
d 0–14	2.47	2.55	2.69	2.37	2.49	2.47	0.097	0.032	0.077	0.49
d 15–28	2.73 ^h,i,j^	2.68 ^h,i^	2.52 ^h^	3.01 ^j^	3.51 ^k^	2.97 ^i,j^	0.148	<0.001	0.006	0.034
d 0–28	2.59	2.61	2.58	2.70	2.87	2.70	0.067	<0.001	0.091	0.25
CW ^e^ (kg)	67.7 ^j,k^	65.3 ^h,i,j^	65.9 ^i,j^	69.8 ^k^	64.4 ^h,i^	62.8 ^h^	1.28	0.42	<0.001	0.027
DP ^f^ (%)	65.4 ^i^	65.6 ^i^	65.6 ^i^	65.7 ^i^	65.2 ^i^	64.2 ^h^	0.421	0.059	0.11	0.028
P2 backfat (mm) ^g^	9.28 ^h,i^	9.07 ^h,i^	8.90 ^h^	11.1 ^j^	9.49 ^h,i^	9.66 ^i^	0.391	<0.001	0.047	0.042

^a^ Control: Control diet; ^b^ Albus 14: fed diet containing 20% albus lupins for 14 days pre-slaughter; ^c^ Albus 28: fed diet containing 20% albus lupins for 28 days pre-slaughter; ^d^ SED for Sex × Feeding strategy; ^e^ CW: carcass weight; ^f^ DP: dressing percentage; ^g^ Carcass weight used as a covariate; ^h–k^ different superscripts within the same row are significantly different.

**Table 5:** Body composition for entire male and immunocastrated male pigs fed three different feeding strategies from 72.3 to 101.1 kg LW (*n* = 12).

	Entire Male			Immunocastrated Male			SED ^e^	*p*-Value		
	Control ^a^	Albus 14 ^b^	Albus 28 ^c^	Control	Albus 14	Albus 28		Sex	Feeding Strategy	Sex × Feeding Strategy
% BMC ^e^	1.88 ^h^	1.82 ^g,h^	1.75 ^f,g^	1.70 ^f^	1.79 ^f,g,h^	1.86 ^h^	0.053	0.28	0.91	0.001
% Lean	82.5	82.9	83.7	79.1	81.7	81.4	1.071	<0.001	0.055	0.33
% Fat	15.6	15.3	14.6	19.2	16.5	16.8	1.083	<0.001	0.056	0.29

^a^ Control: Control diet; ^b^ Albus 14: fed diet containing 20% albus lupins for 14 days pre-slaughter; ^c^ Albus 28: fed diet containing 20% albus lupins for 28 days pre-slaughter; ^d^ SED for Sex × Feeding strategy; ^e^ BMC—bone mineral content; ^d–f^ different superscripts within the same row are significantly different.
